# Delivering the precision oncology paradigm: reduced R&D costs and greater return on investment through a companion diagnostic informed precision oncology medicines approach

**DOI:** 10.1186/s40545-023-00590-9

**Published:** 2023-07-05

**Authors:** Raymond H. Henderson, Declan French, Elaine Stewart, Dave Smart, Adam Idica, Sandra Redmond, Markus Eckstein, Jordan Clark, Richard Sullivan, Peter Keeling, Mark Lawler

**Affiliations:** 1 Patrick G. Johnston Centre for Cancer Research, Queen’s University, Belfast, UK; 2 Queen’s Management School, Queen’s University Belfast, Belfast, UK; 3 Diaceutics PLC, Dataworks at Kings Hall Health and Wellbeing Park, Co Antrim, Belfast, BT9 6GW UK; 4 Salutem Insights Ltd, Clough, Portlaoise, Garryduff, R32 V653 Ireland; 5 Inovalon Inc., 4321 Collington Road, Bowie, MD 20716 USA; 6 Institute of Pathology, University Hospital Erlangen, Erlangen, Germany; 7 Institute of Cancer Policy, School of Cancer Sciences, King’s College London, London, UK

## Abstract

**Background:**

Precision oncology medicines represent a paradigm shift compared to non-precision oncology medicines in cancer therapy, in some situations delivering more clinical benefit, and potentially lowering healthcare costs. We determined whether employing a companion diagnostic (CDx) approach during oncology medicines development delivers effective therapies that are within the cost constraints of current health systems. R&D costs of developing a medicine are subject to debate, with average estimates ranging from $765 million (m) to $4.6 billion (b). Our aim was to determine whether precision oncology medicines are cheaper to bring from R&D to market; a secondary goal was to determine whether precision oncology medicines have a greater return on investment (ROI).

**Method:**

Data on oncology medicines approved between 1997 and 2020 by the US Food and Drug Administration (FDA) were analysed from the Securities and Exchange Commission (SEC) filings. Data were compiled from 10-K, 10-Q, and 20-F financial performance filings on medicines’ development costs through their R&D lifetime. Clinical trial data were split into clinical trial phases 1–3 and probability of success (POS) of trials was calculated, along with preclinical costs. Cost-of-capital (CoC) approach was applied and, if appropriate, a tax rebate was subtracted from the total.

**Results:**

Data on 42 precision and 29 non-precision oncology medicines from 56 companies listed by the National Cancer Institute which had complete data available were analysed. Estimated mean cost to deliver a new oncology medicine was $4.4b (95% CI, $3.6–5.2b). Costs to bring a precision oncology medicine to market were $1.1b less ($3.5b; 95% CI, $2.7–4.5b) compared to non-precision oncology medicines ($4.6b; 95% CI, $3.5–6.1b). The key driver of costs was POS of clinical trials, accounting for a difference of $591.3 m. Additional data analysis illustrated that there was a 27% increase in return on investment (ROI) of precision oncology medicines over non-precision oncology medicines.

**Conclusion:**

Our results provide an accurate estimate of the R&D spend required to bring an oncology medicine to market. Deployment of a CDx at the earliest stage substantially lowers the cost associated with oncology medicines development, potentially making them available to more patients, while staying within the cost constraints of cancer health systems.

**Supplementary Information:**

The online version contains supplementary material available at 10.1186/s40545-023-00590-9.

## Background

The research and development (R&D) costs of developing an oncology medicine are frequently debated, with average cost estimates ranging from $765 million (m) to $4.6 billion (b) (in 2020 US dollars) [1, 2]. Precision oncology medicines require a companion diagnostic (CDx) to guide treatment for patients who will respond to therapy, whereas non-precision oncology medicines involve a “one size fits all” approach with no discriminatory test to distinguish responders from non-responders. Benchmarked against the European Society of Medical Oncology Magnitude of Clinical Benefit Scale (MCBS), the CDx-oncology medicine combination can deliver more clinically meaningful benefit in both curative and non-curative settings [3, 4]. If more complex and potentially more expensive precision oncology medicines are to have a truly global impact, then CDx-guided oncology medicine development and deployment are absolutely essential.

In seeking to reduce R&D costs through earlier patient stratification by a CDx approach, pharmaceutical companies can better manage their oncology pipelines, improve timing of phase transitions into, ultimately, marketing authorizations for CDx-oncology medicines that deliver clinically meaningful benefit, in what is becoming a complex regulatory and competitive environment. Precision medicine has been hailed as a potential breakthrough for cancer patients and a disruptive technology which will potentially lower healthcare costs [3, 5], but studies that deploy health economic analysis to determine the value of a precision medicine approach have been scarce [6, 7]. Reducing R&D cost is not just beneficial for industry, but also provides a better environment for fair pricing and reimbursement on a country-by-country basis.

Our study goal was to determine whether precision oncology medicines are cheaper to bring from R&D to market; a secondary goal was to determine whether precision oncology medicines have a greater return on investment (ROI). The employment of a CDx to specifically target oncology medicines to patient tumours can potentially lead to more effective clinical trial outcomes [8]. The ability to achieve lower R&D costs in the delivery of effective precision oncology medicines provides an opportunity for pharmaceutical companies to invest in a CDx-guided approach that enhances patient access to innovative medicines at a cost that health systems can bear, with the additional benefit to companies of a potential tripling of their market share [9].

## Methods

This is a secondary data analysis of published US government and verified public databases. Oncology-specific medicines are tracked throughout their R&D clinical journey, expenditure, and where applicable their sales.

### Oncology drug identification

Using the US National Cancer Institute (NCI) oncology drug database, we identified all biologics and small molecules registered with Food and Drug Administration’s (FDA) marketing authorization for an oncology indication since the start of the targeted oncology era (1997) until 2020 [10]. Information on each medicine was then extracted from the FDA’s Drugs@FDA database, including original manufacturer (and parent company), date of approval, disease indication, drug type (e.g., biologic or small molecule), CDx employed or not, orphan status, expedited programs (accelerated approval, breakthrough, fast track, or priority review), and whether the medicine was first-in-class [11, 12]. Each medicine was assessed for the intention of requiring a CDx or not for clinical delivery. Those that were developed with the intention of deploying a CDx were considered precision oncology medicines, and those where there was no intention to deploy a CDx were considered non-precision oncology medicines. CAR T-cell therapies, radiopharmaceuticals, and hormonal blockers were excluded, to ensure our comparative dataset of medicines had similar R&D pathways. Start and end dates for clinical trials (phase I, II, and III) for each medicine were determined by utilizing the Clinicaltrials.gov database capturing duration and the number of enrolees in the R&D process for each oncology medicine; dates of these clinical trials were confirmed with the U.S. Securities and Exchange Commission (SEC) filings from the Electronic Data Gathering, Analysis, and Retrieval (EDGAR) system database, or the medicine manufacturer’s annual reports [13, 14].

### Data extraction

Information from 56 pharmaceutical companies were evaluated through the SEC EDGAR database to obtain R&D budgets and sales data for each oncology medicine via 10-K and 10-Q (for US public companies) or 20-F filings (for non-US companies listed in USA); these were supplemented where possible with the medicine manufacturer (or parent company) annual reports. The quality of data for each medicine was graded as in Box 1 [15].

**Box 1:**
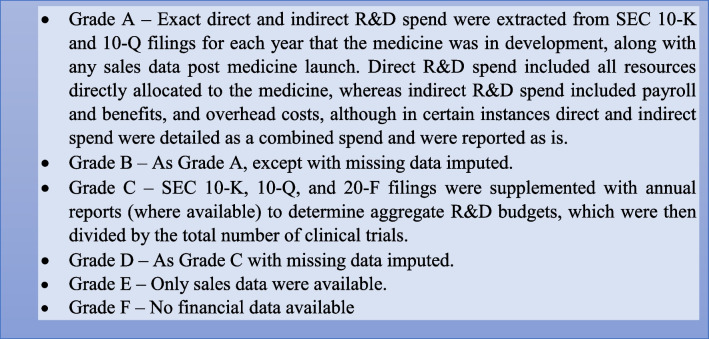
Data grading system

A total of 11.5% of grade A and B combined had missing entries and 16.1% of grade C and D combined also had missing financial data. The SEC database was searched using the generic, brand, or compound name of the medicine; spend was tracked via the company’s financial statements from first-in-human trial to launch, and sales data were tracked post-launch. Using spend accrued for both SEC and modelling data which had been split into phase I, II, and III trials, the cost of success was calculated, employing clinical trial success rates from Wong et al. [8] (see Additional file 1: Table S1).

### Costing method

Initial R&D medicine spend captured preclinical, phase I, II, and III spend; probability of success (POS), the cost of capital (CoC), and any tax credits subtracted were then added. First, we imputed 42.9% of the total R&D spend from SEC filings for each medicine to account for preclinical expenses, following DiMasi et al.’s (2016) calculation of preclinical to R&D cost ratio [16]. Secondly, to rationalize attrition rates, we divided the total R&D investment for each phase of medicine development by its POS factor (see Additional file 1: Table S1), which was dependent on whether or not a CDx was deployed throughout the medicine’s clinical development. Thirdly, a 10.5% cost of capital was used to account for the time value of money following DiMasi et al. [16]. Fourthly, any tax credits or rebates were applied to the initial R&D investment and this amount was subtracted from the total [17–20]. Lastly, all R&D spend, and sales data were converted to US dollars and inflation adjusted to 2020 using the Campbell and Cochrane Economics Methods Group/Evidence for Policy and Practice Information and Coordinating Centre Cost Converter [21].

### Statistical analysis

The mean and median of R&D financing was calculated along with means for company age, total revenue, trial duration, trial enrolees, medicine sales, and ROI, and the median of R&D investment as a proportion of total revenue. We then split the sample into precision and non-precision oncology medicines and recalculated the means and medians. We narrowed the calculations further to precision and non-precision oncology medicines based on data quality (Grade A to Grade E) as described above. ROI was calculated by dividing the net value of investment divided by the cost of investment as follows:

$$\mathrm{ROI}=\frac{\mathrm{FVI}-\mathrm{IVI}}{\mathrm{IVI}}\times 100\%,$$

where FVI = final value of investment (medicine sales), IVI = initial value of investment (final R&D cost).

Confidence intervals (CIs) for our final R&D spend were calculated by a bootstrapping resampling and replacement (1000 iterations) approach. The t-test was used to identify statistically significant differences in sample data means overall between precision and non-precision oncology medicines; between Grade A and B precision and non-precision oncology medicines; and between Grade C and D precision and non-precision oncology medicines. All statistical tests were 2-tailed and used a type I error rate of 0.05. The data were analysed using Stata version 16 (StataCorp).

## Results

**Fig. 1:**
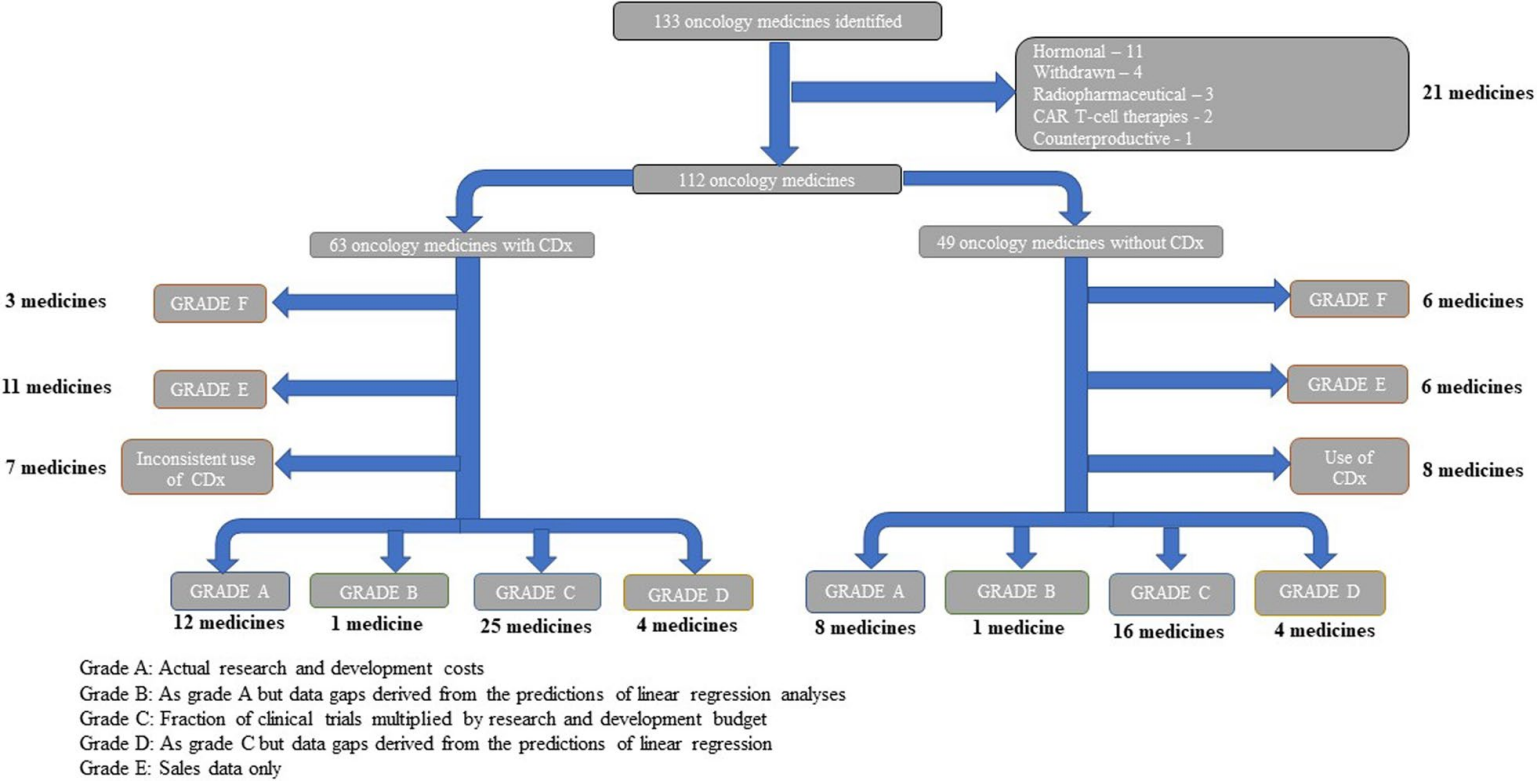
Flowchart of identified studies and data grading. Flowchart showing the identification, screening and grading of oncology medicines used to estimate the difference in the estimated R&D spend between precision and non-precision oncology medicines. From the dataset, CAR T-cell therapies, radiopharmaceuticals, and hormonal blockers, etc., were excluded and the remaining 112 oncology medicines split into precision and non-precision oncology medicines. Oncology medicines without clinical trial data were removed (Grade E and F). Finally, precision medicines which did not consistently use a CDx in their trials were excluded (*n* = 7), as were non-precision medicines which did use a CDx in their clinical trials (*n* = 8). *CDx* companion diagnostic, *R&D* research and development

The FDA approved 133 oncology medicines between 1997 and 2020. After the removal of CAR T-cell therapies and hormonal blockers (*n* = 21), there were 112 medicines with an oncology indication detailed in SEC filings, annual reports, and/or sourced from Clinicaltrials.gov (Fig. 1). Of this dataset (*n* = 112), 56.3% (*n* = 63) were initially classed as precision oncology medicines and 43.7% (*n* = 49) were classed as non-precision oncology medicines.

On further analysis, 15 medicines could not be entered into the financial analysis because of inconsistent application of the CDx approach in certain parts of the R&D processes (both precision and non-precision pathways). Medicines with either no financial data (Grade F; *n* = 9) or sales data only (Grade E; *n* = 17) were also excluded from the final analytical datasets (see Additional file 1: Table S3).

Of the final set of 71 cancer medicines for detailed analysis, 21 were assessed as being high quality ((Grade A and B) and 59 as medium quality (Grade C and D), see Fig. 1).

### Dataset characteristics

Table 1 reports statistics for our datasets, split by precision (*n* = 42) and non-precision (*n* = 29) oncology medicines. Focusing on the precision oncology medicine dataset, testing for *HER2*^+^ was the most frequent CDx employed for precision oncology medicines (*n* = 8), other CDxs for precision oncology medicines included testing for *BRAF* (*n* = 4) *ALK*, *EGFR, HER2*^*−*^, and *PD-L1 *(n = 3)* BRCA*, *FLT3*^+^, *NTRK*, and *RET *(*n* = 2) and one each for the remaining CDxs (*n* = 1). The vast majority of precision oncology medicines were small molecules (e.g., tyrosine kinase inhibitors) rather than biologics (e.g., monoclonal antibodies), whereas amongst the non-precision oncology medicines, biologics and small molecules were roughly equivalent in number. The proportion of orphan medicines in both the precision and non-precision datasets were almost equivalent, while there was a greater proportion of first-in-class amongst the non-precision oncology medicine dataset, while more precision oncology medicines have benefited from expedited programs (accelerated approval, breakthrough, fast track, or priority review). The numbers of precision oncology medicines gaining FDA approval in our dataset have outpaced non-precision oncology medicines.

**Table 1:** Characteristics and gene targets for oncology medicines approved by FDA

	CDx (*N* = 42)	Non-CDx (*N* = 29)
CDxs^a^		
ALK	3 (7.1)	–
BCR-ABL+	1 (2.4)	–
BCR-ABL−	1 (2.4)	–
BRAF	4 (11.9)	–
BRCA	2 (4.8)	–
EGFR	3 (7.1)	–
EZH2	1 (2.4)	–
FGFR	1 (2.4)	–
FLT3+	2 (4.8)	–
HER2+	8 (19.0)	–
HER2−	3 (7.1)	–
IDH1	1 (2.4)	–
IDH2	1 (2.4)	–
MET	1 (2.4)	–
NTRK	2 (4.8)	–
PDGFRA	1 (2.4)	–
PD-L1	3 (7.1)	–
PIK3CA	1 (2.4)	–
RAS	1 (2.4)	–
RET	2 (4.8)	–
ROS	1 (2.4)	–
No CDx	–	29 (100.0)
Small molecule	31 (73.8)	12 (41.4)
Biologic	11 (26.2)	17 (58.6)
Orphan drug	28 (66.7)	20 (69.0)
First in class	15 (35.7)	15 (51.7)
Expedited program	40 (95.2)	24 (82.8)
Approval dates		
1997–2002	1 (2.4)	2 (6.9)
2003–2008	4 (9.5)	2 (6.9)
2009–2014	7 (16.7)	9 (31.0)
2015–2020	30 (71.4)	16 (55.2)

*ALK* anaplastic lymphoma kinase, *BCR-ABL1* breakpoint cluster region and Abelson murine leukemia, *BRAF−* proto-oncogene B-Raf, *BRCA* breast cancer; *precision* companion diagnostic, *EGFR−* epidermal growth factor receptor, *EZH2* enhancer of zeste homolog 2; *FGFR* fibroblast growth factor receptor, *FLT3* fms like tyrosine kinase 3, *HER2* human epidermal growth factor receptor 2, *IDH* isocitrate dehydrogenase, *MET* MET gene, *n* number; *NTRK* neurotrophin receptor tyrosine kinase, *PDGFRA* platelet-derived growth factor receptor A, *PD-L1* programmed death ligand 1, *PIK3CA* phosphatidylinositol-4,5-bisphosphate 3-kinase, catalytic subunit alpha, *RAS* rat sarcoma virus oncogene, *RET* rearranged during transfection, *ROS* c-ros oncogene

^a^Proportions amongst CDxs may not round to 100% as some drugs have multiple CDxs

### R&D spend

After accounting for the cost of failure (clinical trial attrition rates) (Table S1, supplemental), the estimated mean cost to launch a new oncology medicine to market at a capitalized rate of 10.5% [17] was (all in USD) $4432 million (m) (95% CI, $3624–5240 m); the estimated median was $3160 m (95% CI, $2646–3872 m) (Table 2).

**Table 2:** Median and mean research and development spend including probability of success, cost of capital, and tax rebates

Category and type	Sample *N*	Expenditure in US$, millions				Precision vs non-precision
		Mean	CI, 95%	Median	CI, 95%	*p-*value
All (inconsistent use of CDx))	86	4432.1	(3624.4–5239.7)	3160.4	(2645.9–3872.2)	
All (CDx use consistent)	71	3973.5	(3318.0–4816.4)	2932.6	(2160.3–3438.7)	
All precision	42	3530.6	(2729.2–4510.9)	2641.3	(2110.1–3545.7)	
All non-precision	29	4614.9	(3532.8–6054.4)	3505.7	(2736.8–5875.3)	0.17
A&B precision	13	2475.0	(1730.7–3483.6)	2110.1	(829.5–2984.0)	
A&B non-precision	9	6124.0	(3472.8–10,007.6)	2891.8	(2645.9–10,749.0)	0.03
C&D precision	29	4003.7	(2917.2–5422.0)	2932.6	(2018.9–3872.2)	
C&D non-precision	20	3935.8	(3040.7–4898.4)	3787.6	(3438.7–5881.6)	0.94

Legend: CDx: companion diagnostic; CIs – confidence intervals; N: number

The estimated mean R&D costs for precision oncology medicines was $3531 m (95% CI, $2729–4511 m) and the estimated median was $2641 m (95% CI, $2110–3546 m), while non-precision oncology medicines had an estimated mean of $4615 m (95% CI, $3533–6054 m), with an estimated median of $3506 m (95% CI, $2737–5875 m), (*p* = 0.17, NS).

**Fig. 2:**
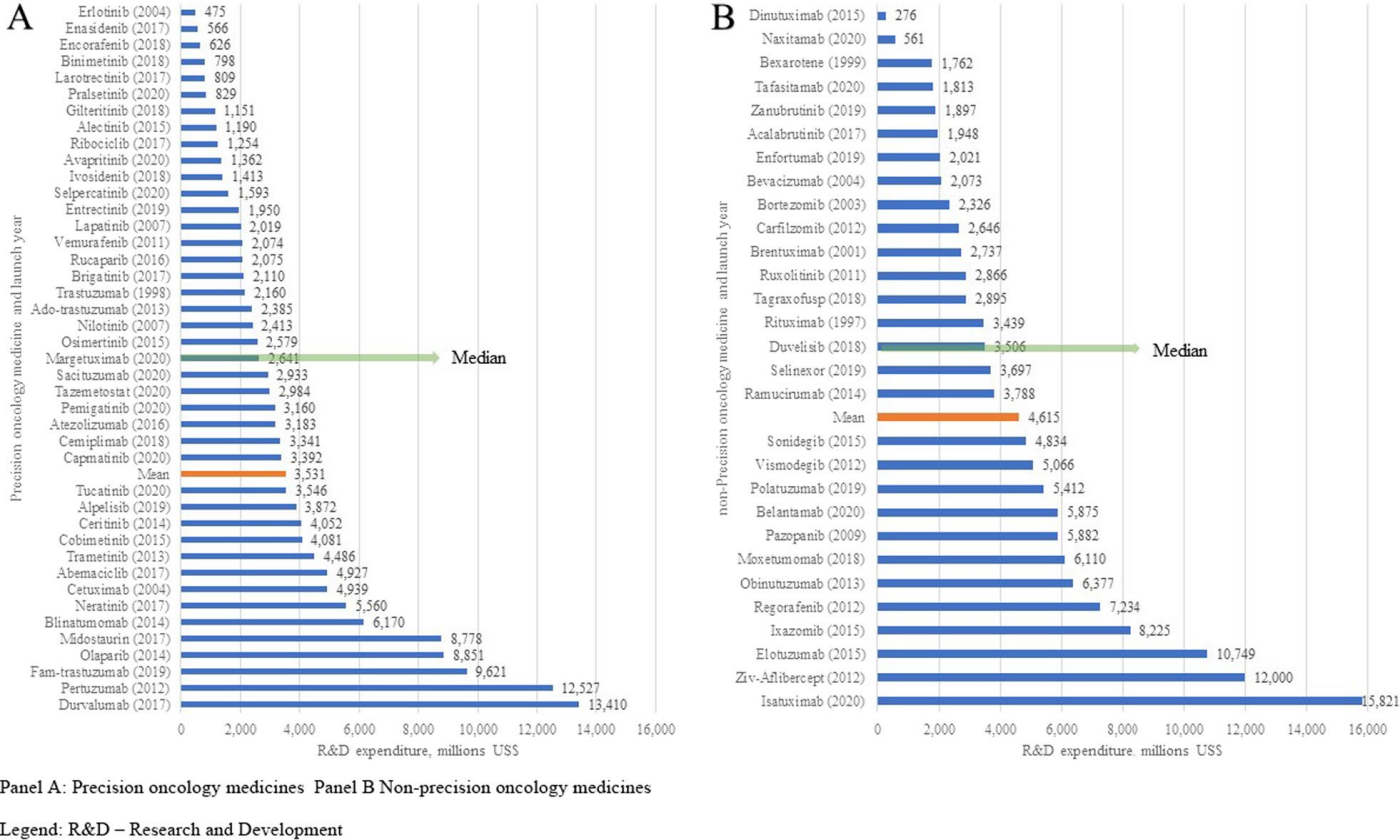
Estimated research and development expenditures of oncology medicines. **A** Precision oncology medicines. Panel B Non-precision oncology medicines. *R&D* research and development

Focusing the same analysis on the higher quality Grade A and B data, the estimated mean for precision oncology medicines was reduced to $2475 m (95% CI, $1731–3484 m) and the estimated median dropped to $2110 m (95% CI, $830–2984 m); for non-precision oncology medicines the estimated mean increased to $6124 m ($3473–10,008 m) and the estimated median decreased to $2892 m (95% CI, $2895-$8709 m) (*p* = 0.03) (Table 2). Figure 2 shows estimates for each of the 42 precision oncology medicines, which ranged from $475 m for erlotinib to $13,410 m for durvalumab, and for the 29 non-precision oncology medicines, ranging from $276 m for dinutuximab to $15,821 m for isatuximab.

The initial R&D costs for precision versus non-precision oncology medicines were $767.5 m versus $774.2 m, a difference of $6.7 m, whereas the POS costs for precision versus non-precision oncology medicines were $1486.6 m versus $2077.9, a difference of $591.3 m. These figures demonstrate that the increased POS associated with a CDx in clinical trials is the key driver of costs, as the employment of a CDx approach increases POS versus a non CDx approach by a factor of 2.5 (see Additional file 1: Fig. S1). [8]

Table 3 shows additional data collated from SEC filings, annual reports, and Clinicaltrials.gov. While not statistically significant, it took marginally more time to develop a precision oncology medicine than a non-precision oncology medicine. From a clinical trial perspective, there were 24% more trial enrolees for precision oncology medicines. While not statistically significant, the overall ROI was 25% greater for non-precision oncology medicines compared to precision oncology medicines. Based on our data, precision oncology medicines currently have a market share of 51.4% of annual sales compared to non-precision oncology medicines (difference not statistically significant).

**Table 3:** Aggregated trial and sales data

	Precision, *n*		Non-precision, *n*		*p*-value
Duration (years)	6.7	42	6.5	29	0.74
Enrolees	2285	40	1838	26	0.41
Total sales (m US$)	5155.8	39	8257.6	27	0.46
Drug sales/year (m US$)	607.6	39	575.0	27	0.90
R&D spend (m US$)	3530.6	42	4614.9	29	0.17
Total sales—R&D spend (m US$)	1499.5	39	3545.2	27	0.64
SEC ROI	148.9%	39	199.5%	27	0.79
SEC ROI (1997–2015)^a^	551.3%	14	435.1%	15	0.77

*m* millions, *n* number, *R&D* research and development, *ROI* return on investment, *SEC* Securities and Exchange Commission

^a^Analysis restricted from 1997 to 2015 to allow time for drug sales to accrue

**Fig. 3:**
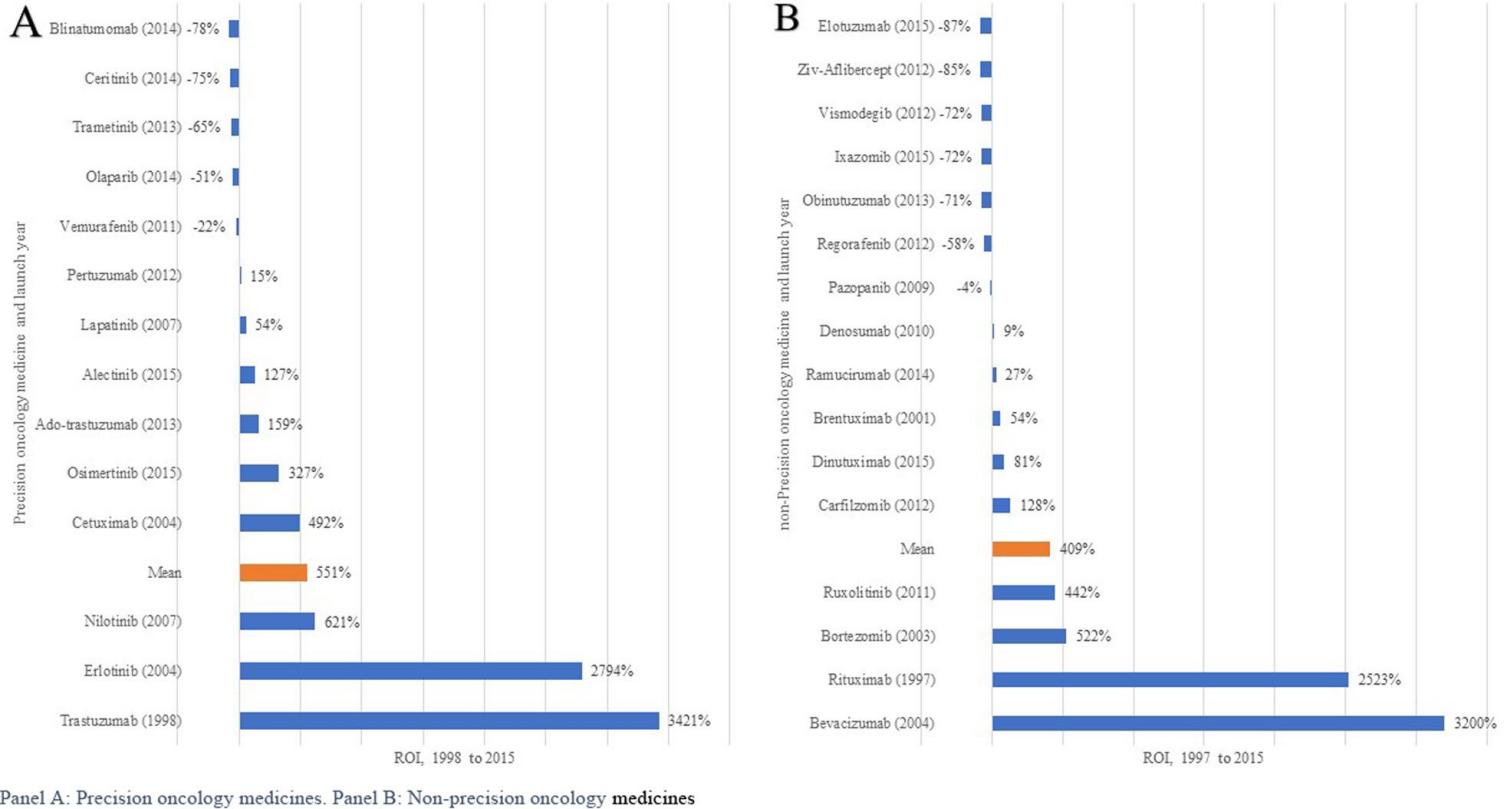
Return on investment of precision and non-precision oncology medicines, restricted from 1997 to 2015. **A** Precision oncology medicines. **B** Non-precision oncology medicines

However, most of the precision oncology medicines have been launched in the last five years compared to non-precision oncology medicines (see Additional file 1: Fig. S2), not allowing sufficient time to accrue optimal medicines sales (Table 3) and thus skewing the ROI calculated above. To address this important issue, a different ROI analysis was conducted that was restricted to medicines launched from 1997 to 2015 to allow for parity in both the number of medicines analysed and in marketing time. Figure 3 shows estimates for precision oncology medicines (*n* = 14) and non-precision oncology medicines (*n* = 15), with a 27% increase in profitability for precision oncology medicines over non-precision oncology medicines.

## Discussion

Generation of this dataset and its interpretation represent the most comprehensive and unique analyses of the R&D costs of oncology medicines to date derived from company filings (see Additional file 1: Table S3). Data for each oncology medicine were evaluated using the same approach, however certain data were missing in the Grade B dataset (11.5% of grade A and B data combined) which primarily occurred for larger pharmaceutical companies which had taken over smaller companies. A total of 16.1% of the Grade C and D combined were Grade D financial data, with missing entries due to R&D taking place before the SEC EDGAR system came online, or takeovers by larger pharmaceutical firms applying less transparent accounting. Nevertheless, the data are reported from 1997 to 2020, all sourced from SEC filings and annual reports, their validity and accuracy have been crosschecked and confirmed. [2]

We employed a cost of capital at 10.5% in this study, consistent with previous studies (Chit et al., DiMasi et al., Wouters et al. [2, 16, 22]). Our total R&D spend to develop an oncology medicine has a mean and median similar to those derived by Wouters et al. (2020) at $4635.4 m and $2879.8 m, respectively, but higher than the figures derived by Prasad and Mailankody (2017) which had a mean of $764.8 m and median of $688 m; the former employed a cost of capital at 10.5% like ours, while the later utilized a cost of capital at 7% [1, 2]. Prasad and Mailankody’s paper was highlighted in a systematic review as applying the lowest cost of capital amongst all studies performed and has been criticized for this bias [23].

While the World Health Organization (WHO) has requested more clarity in the publication of R&D costs, to assist in price negotiations for medicines globally, challenges remain in capturing accurate data, while resolutions to ensure more transparency in reporting R&D spend have been diluted after opposition from Germany, Japan, Switzerland, the United Kingdom, and the United States [24]. Meanwhile, the auditing arm of the SEC, the Public Company Accounting Oversight Board (PCAOB), which acts as a watchdog, has come under criticism from investor groups for not enforcing its own guidance, as to the reliability of firm’s financial disclosures, especially for larger firms [25, 26].

R&D spend needs to be recovered by industry to provide an incentive for discovery and commercialization of precision medicine; however, for health systems to be able to make well-informed decisions on healthcare policy, they need to know the relevant global R&D spend that has occurred. At present, accounting standards allow companies to present R&D spend in vastly different ways, making standardization of R&D spend difficult. As a result, prior research has focused mostly on estimating the R&D spend, rather than on the actual figures. Rigorous adherence to transparent accounting standards for R&D budgets across companies would, based on our study, lead to an affirmation of the precision approach to oncology medicine development, with its dual value of cutting costs and potentially benefitting greater numbers of patients. Our study has found that, despite progress being achieved, many so-called precision oncology medicines do not have a CDx integrated into their development pathway.

Previous analyses have indicated that oncology is the most expensive R&D medicines domain, and companies’ strategies are predicated on driving premium pricing, i.e. what the markets will bear, commercial approaches that threaten to undermine healthcare budgets [2, 27, 28]. Our data for oncology medicines from 56 companies over a 24-year period suggest that in the case of precision oncology medicines, this need not be the case. We demonstrate that it costs over $1 billion more in R&D spend to develop an oncology medicine that is not guided through clinical trials with a CDx, compared to a precision oncology approach. Thus, CDx-guided precision oncology approaches appear to offer better value to the key stakeholders; payers, patients and the pharmaceutical industry.

Our study provides compelling evidence concerning the pricing of precision oncology medicines. Our data indicate that while the R&D associated with developing an oncology medicine requires substantial investment, the deployment of a CDx strategy from the start of the R&D process to help guide treatment decisions offers substantial opportunities to deliver precision oncology medicines with greater clinical benefit, but also at a price that is more affordable than non-precision oncology medicines to health systems, and most importantly to patients. Moving towards a precision oncology CDx-guided approach can deliver health benefits at a potentially affordable cost, including in the development phase, lowering expensive clinical trial attrition rates, and sparing unselected patients those treatments that are ineffective and may have significant side effects [29, 30].

The implications of this study for drug discovery, market access, health outcomes, health and industry policy are far reaching. The increased success of precision oncology medicine’s FDA approval rates not only helps to reduce R&D spend, but also reinforces the rationale for targeting the underlying genomic mechanisms of oncogenesis [8, 31]. Decreased R&D spend and increased effectiveness of precision oncology medicines will likely accelerate the reimbursement process and also require a rethink of market access strategies [3, 32–34].

The realization by the pharmaceutical industry that precision oncology medicines are less expensive and more effective is leading to numerous precision oncology medicines entering the marketplace, this will present challenges to decision-makers regarding equitable treatment provision for all patients in all regions [35]. Finally, the pharmaceutical industry generates $1.25 trillion globally with a 5% growth rate annually. With advances in artificial intelligence and -omic technologies precision medicine sits at the nexus of the fourth industrial revolution, its integration into healthcare systems will benefit not only patients but also the industry itself [36, 37]. Policy makers should therefore revisit the fragmented policy landscape of precision medicine to create the regulatory conditions necessary to supporting precision medicine activities and capitalize on its benefits [38].

We hypothesized in 2013 that precision medicine could deliver better value for both private and public stakeholders [39]; this detailed study of the initial wave of precision oncology medicines in the marketplace suggests that the industry should revisit the commercial model for precision medicines and their associated diagnostic/prognostic tests. The R&D savings that can be accrued by this approach should allow pharmaceutical companies to adopt new business models that not only empower reinvestment in robust tumour testing (to help serve the needs of the two-thirds of patients not currently treated with a precision oncology approach) [9], but also to ensure equity-based pricing systems, as well as developing precision oncology medicines that deliver significant, clinically meaningful benefit.

## Limitations

There are several limitations in this study. Firstly, the quality of the data was only high (Grade A and B) in 19.6% of cases (mainly small US companies); 57.2% of the data were of medium quality (Grade C and D, mainly non-US companies and some large US companies), while 23.2% of data were low-quality (Grade E and F), sourced from both large US companies and non-US companies. Our analysis is only statistically significant when restricted to small US companies with rigorous and transparent accounting standards, when conclusions relating to the decreased R&D cost of a precision oncology medicine are sound. Generally, we have demonstrated on average it costs almost $1.1 billion more to bring a non-precision oncology medicine through the cancer drug development process, compared to a precision oncology medicine, but although trending, this difference is not statistically significant.

Secondly, the figures presented may underestimate the final R&D cost of an oncology medicine, as clinical trials can continue after a medicine has been approved by the FDA.

## Conclusion

This study puts forward an evidence-informed estimation of the R&D spend associated with bringing an oncology medicine through R&D and clinical trials to market. The intelligence generated in this study indicates that the deployment of a CDx at the earliest stage substantially lowers the cost associated with oncology medicine development, potentially making it available to more patients, while staying within the cost constraints of cancer health systems. We have reached a crucial inflection point, which requires a flexible CDx development framework so that patients can truly benefit from a precision oncology approach, while at the same time ensuring that R&D spend in oncology medicine development overall is affordable to health systems.

## Supplementary Information

**Additional file 1**: **Table S1: **Probability of success of precision and non-precisiononcology medicines. **Table S2**:Oncology medicines, their CDxs, and clinical indications. **Table S3**:Oncology medicines, their clinical trials, cost, and revenue. **FigureS1**: Difference in research and development spendbetween precision and non-precision oncology medicines. **FigureS2**: Number of precision versus non-precision medicines launched from our sample.

## Data Availability

Derived data supporting the findings of this study are available in the Additional file.

## Abbreviations

b: Billion
CDx: Companion diagnostic
CI: Confidence interval
CoC: Cost of capital
EDGAR: Electronic data gathering, analysis, and retrieval
FDA: Food and Drug Administration
m: Million
MCBS: Magnitude of Clinical Benefit Scale
NCI: National Cancer Institute
POS: Probability of success
R&D: Research and development
ROI: Return on investment
SEC: Securities Exchange Commission
USD: United States dollars
WHO: World Health Organization
