# Supplementary material for: Delivering the precision oncology paradigm: reduced R&D costs and greater return on investment through a companion diagnostic informed precision oncology medicines approach
Source: J Pharm Policy Pract. 2023 Jul 5;16:84. doi: 10.1186/s40545-023-00590-9 (PMC10320864; doi:10.1186/s40545-023-00590-9)
Supplement: Supplementary file 1 — Additional file 1: Table S1: Probability of success of precision and non-precisiononcology medicines. Table S2:Oncology medicines, their CDxs, and clinical indications. Table S3:Oncology medicines, their clinical trials, cost, and revenue. FigureS1: Difference in research and development spendbetween precision and non-precision oncology medicines. FigureS2: Number of precision versus non-precision medicines launched from our sample. [file 40545_2023_590_MOESM1_ESM.docx]

**Delivering the precision oncology paradigm – reduced R&D costs and greater return on investment through a companion diagnostic informed precision oncology medicines approach**

**Additional file 1**

**Table S1: Probability of success of precision and non-precision oncology medicines**

|  | Probability of Success (%)* | |
| --- | --- | --- |
| Trial | **precision** | **non-precision** |
| phase I | 33.5 | 26.3 |
| phase II | 25.8 | 16.2 |
| phase III | 40.8 | 33.6 |

Legend:*Data from a sample of over 27,000 oncology clinical trials, performed between 2006 and 2015, were used to inform an algorithm which calculated the probability of success in each phase of the trial with and without a companion diagnostic, from Wong *et al*. ^1^

**Table S2: Oncology medicines, their CDxs, and clinical indications**

| Parent/Company | Generic name | Brand name | CDx | Indications | Number of indications (precision)* | Precision medicine? |
| --- | --- | --- | --- | --- | --- | --- |
| Eli Lilly | Abemaciclib | Verzenio™ | HR+, HER2- | BC | 1 (1) | Yes |
| AstraZeneca | Acalabrutinib | Calquence® |  | CLL | 1 (0) | No |
| Roche/Genentech | Ado-trastuzumab | Kadcyla® | HER2+ | BC | 1 (1) | Yes |
| Boehringer | Afatinib | Gilotrif® | EGFR | NSCLC | 1 (1) | Yes |
| Roche/Chugai | Alectinib | Alecensa® | ALK | NSCLC | 1 (1) | Yes |
| Novartis | Alpelisib | Piqray® | HR+, HER2-, PIK3CA | BC | 1 (1) | Yes |
| Roche | Atezolizumab | Tecentriq® | PD-L1 | HCC, NSCLC, SCLC, TNBC, and UC | 5 (3) | Yes |
| Blueprint | Avapritinib | Ayvakit™ | PDGFR | AdvSM and GIST | 2 (1) | Yes |
| Merck~Pfizer | Avelumab | Bavencio® |  | MCC, RCC, and UC | 2 (0) | No |
| Pfizer | Axitinib | Inlyta® |  | RCC | 1 (0) | No |
| GSK | Belantamab | Blenrep |  | MM | 1 (0) | No |
| Genentech | Bevacizumab | Avastin® |  | BC, CC, CRC, NSCLC, OC, and RCC | 6 (0) | No |
| Ligand | Bexarotene | Targretin® |  | CTCL | 1 (0) | No |
| Pfizer/Array Biopharma | Binimetinib | Mektovi® | BRAF V600 | Melanoma | 1 (1) | Yes |
| Amgen | Blinatumomab | Blincyto® | Ph- | ALL | 1 (1) | Yes |
| Millennium | Bortezomib | Velcade® |  | MM and MCL | 2 (0) | No |
| Pfizer/Wyeth | Bosutinib | Bosulif® | Ph+ | CML | 1 (1) | Yes |
| Seattle Genetics | Brentuximab | Adcetris® |  | CTCL and HL | 2 (0) | No |
| Takeda/Ariad | Brigatinib | Alunbrig™ | ALK, EGFR | NSCLC | 1 (1) | Yes |
| Sanofi | Cabazitaxel | Jevtana® |  | PrC | 1 (0) | No |
| Exelixis | Cabozantinib | Cometriq® |  | DTC, HCC, and RCC | 3 (0) | No |
| Incyte | Capmatinib | Tabrecta® | MET | NSCLC | 1 (1) | Yes |
| Onyx | Carfilzomib | Kyprolis® |  | MM | 1 (0) | No |
| Regeneron | Cemiplimab | Libtayo® | PD-L1 | BCC, CSCC, and NSCLC | 3 (1) | Yes |
| Novartis | Ceritinib | Zykadia™ | ALK | NSCLC | 1 (1) | Yes |
| ImClone | Cetuximab | Erbitux® | RAS | CRC and HNC | 2 (1) | Yes |
| Exelixis | Cobimetinib | Cotellic™ | BRAF V600 | MM | 1 (1) | Yes |
| Bayer | Copanlisib | Aliqopa™ |  | FL | 1 (0) | No |
| Pfizer | Crizotinib | Xalkori® | ALK, ROS | NSCLC | 1 (1) | Yes |
| GSK | Dabrafenib | Tafinlar® | BRAF V600 | MM and NSCLC | 2 (2) | Yes |
| Pfizer | Dacomitinib | Vizimpro® | EGFR | NSCLC | 1 (1) | Yes |
| Johnson & Johnson | Daratumumab | Darzalex™ |  | MM | 1 (0) | No |
| BMS | Dasatinib | Sprycel® | Ph+ | ALL and CML | 2 (2) | Yes |
| United Therapeutics | Dinutuximab | Unituxin™ |  | Neuroblastoma | 1 (0) | No |
| AstraZeneca | Durvalumab | Imfinzi™ | PD-L1 | NSCLC, SCLC, and UC | 3 (3) | Yes |
| Infinity | Duvelisib | Copiktra™ |  | CLL, FL, and SLL | 3 (0) | No |
| Abbvie~BMS | Elotuzumab | Empliciti™ |  | MM | 1 (0) | No |
| Agios | Enasidenib | Idhifa® | IDH2 | AML | 1 (1) | Yes |
| Pfizer/Array Biopharma | Encorafenib | Braftovi® | BRAF V600 | CRC and melanoma | 2 (2) | Yes |
| Astellas | Enfortumab | Padcev™ |  | UC | 1 (0) | No |
| Roche/Ignyta | Entrectinib | Rozlytrek™ | ROS1, NTRK | NSCLC and NTRK tumors | 2 (2) | Yes |
| Johnson & Johnson | Erdafitinib | Balversa™ | FGFR2/3 | UC | 1 (1) | Yes |
| Roche/OSI | Erlotinib | Tarceva® | EGFR | NSCLC and PaC | 2 (1) | Yes |
| Novartis | Everolimus | Afinitor® | HER2- | BC, NT (Pa), NT (GI or L), and RCC | 4 (1) | Yes |
| Daiichi Sankyo | Fam-trastuzumab | Enhertu® | HER2+ | BC | 1 (1) | Yes |
| BMS | Fedratinib | Inrebic® |  | MF | 1 (0) | No |
| AstraZeneca | Gefitinib | Iressa® | EGFR | NSCLC | 1 (1) | Yes |
| Astellas | Gilteritinib | Xospata® | FLT3 | AML | 1 (1) | Yes |
| Pfizer | Glasdegib | Daurismo™ |  | AML | 1 (0) | No |
| Pharmacyclics | Ibrutinib | Imbruvica® |  | CLL, MCL, and WM | 3 (0) | No |
| Gilead | Idelalisib | Zydelig® | TP53 | CLL and FL | 2 (1) | Yes |
| Novartis | Imatinib | Gleevec® | Kit, Ph+, PDGFR | ALL, CEL and HES, CML, DFSP, GIST, and MDS/MPD | 6 (5) | Yes |
| Pfizer | Inotuzumab | Besponsa® |  | ALL | 1 (0) | No |
| BMS | Ipilimumab | Yervoy® | PD-L1, MSI-H | CRC (MSI-H), MPM, NSCLC, and OSCC | 4 (2) | Yes |
| Sanofi | Isatuximab | Sarclisa® |  | MM | 1 (0) | No |
| Agios | Ivosidenib | Tibsovo® | IDH1 | AML | 1 (1) | Yes |
| Takeda | Ixazomib | Ninlaro® |  | MM | 1 (0) | No |
| GSK | Lapatinib | Tykerb® | HER2+ | HER2^+^ tumors | 1 (1) | Yes |
| Loxo | Larotrectinib | Vitrakvi® | NTRK | NTRK tumors | 1 (1) | Yes |
| Eisai | Lenvatinib | Lenvima® |  | DTC, EC, and HCC | 3 (0) | No |
| Pfizer | Lorlatinib | Lorbrena® | ALK | NSCLC | 1 (1) | Yes |
| MacroGenics | Margetuximab | Margenza® | HER2+ | BC | 1 (1) | Yes |
| Novartis | Midostaurin | Rydapt® | FLT3 | AML and ASM | 2 (1) | Yes |
| Kyowa Kirin | Mogamulizumab | Poteligeo® |  | MF or SS | 1 (0) | No |
| AstraZeneca | Moxetumomab | Lumoxiti™ |  | HCL | 1 (0) | No |
| Y-mAbs Therapeutics | Naxitamab | Danyelza^TM^ |  | Neuroblastoma | 1 (0) | No |
| Puma Biotechnology | Neratinib | Nerlynx™ | HER2- | BC | 1 (1) | Yes |
| Novartis | Nilotinib | Tasigna® | Ph+ | CML | 1 (1) | Yes |
| Tesaro | Niraparib | Zejula™ |  | OC | 1 (0) | No |
| BMS | Nivolumab | Opdivo® | PD-L1, MSI-H | CRC (MSI-H), cHL, GEJC, Melanoma, MPM, NSCLC, OSCC, SCCHN, and UC | 8 (4) | Yes |
| Roche/Genentech | Obinutuzumab | Gazyva® |  | CLL and FL | 2 (0) | No |
| AstraZeneca | Olaparib | Lynparza™ | BRCA 1/2 | BC, OC, PaC, and PrC | 4 (4) | Yes |
| AstraZeneca | Osimertinib | Tagrisso™ | EGFR | NSCLC | 1 (1) | Yes |
| Pfizer | Palbociclib | Ibrance® | HR+, HER2- | BC | 1 (1) | Yes |
| Amgen | Panitumumab | Vectibix® | RAS | CRC | 1 (1) | Yes |
| GSK | Pazopanib | Votrient® |  | RCC and STS | 2 (0) | No |
| Merck | Pembrolizumab | Keytruda® | PD-L1, MSI-H | CC, cHL, EC, HNSCC, Melanoma, MSI-H, NSCLC, OeC, RCC, TNBC, and UC | 11 (6) | Yes |
| Incyte | Pemigatinib | Pemazyre® | FGFR2 | Cholangiocarcinoma | 1 (1) | Yes |
| Roche/Genentech | Pertuzumab | Perjeta® | HER2+ | BC | 1 (1) | Yes |
| Roche/Genentech | Polatuzumab | Polivy™ |  | DLBCL | 1 (0) | No |
| Ariad | Ponatinib | Iclusig® | Ph+ | ALL and CML | 2 (2) | Yes |
| Blueprint | Pralsetinib | Gavreto® | RET | NSCLC | 1 (1) | Yes |
| Eli Lilly | Ramucirumab | Cyramza® |  | CRC, GC, HCC, and NSCLC | 4 (0) | No |
| Bayer | Regorafenib | Stivarga® |  | CRC, GIST, and HCC | 3 (0) | No |
| Novartis | Ribociclib | Kisqali® | HR+, HER2- | BC | 1 (1) | Yes |
| Deciphera | Ripretinib | Qinlock |  | GIST | 1 (0) | No |
| Genentech | Rituximab | Rituxan® |  | CLL and NHL | 2 (0) | No |
| Clovis | Rucaparib | Rubraca™ | BRCA 1/2 | OC | 1 (1) | Yes |
| Incyte | Ruxolitinib | Jakafi® |  | MF and PV | 2 (0) | No |
| Gilead/Immunomedics | Sacituzumab | Trodelvy® | HR-, HER2- | TNBC | 1 (1) | Yes |
| Karyopharm | Selinexor | Xpovio™ |  | MM | 1 (0) | No |
| Eli Lilly/Loxo | Selpercatinib | Retevmo® | RET | MTC and NSCLC | 2 (2) | Yes |
| AstraZeneca | Selumetinib | Koselugo® |  | NF1 | 1 (0) | No |
| Johnson & Johnson | Siltuximab | Sylvant® |  | MCD | 1 (0) | No |
| Novartis | Sonidegib | Odomzo® |  | BCC | 1 (0) | No |
| Bayer | Sorafenib | Nexavar® |  | DTC, HCC, and RCC | 3 (0) | No |
| Pfizer | Sunitinib | Sutent® |  | GIST, pNET, and RCC | 3 (0) | No |
| MorphoSys | Tafasitamab | Monjuvi® |  | DLBCL | 1 (0) | No |
| Stemline | Tagraxofusp | Elzonris™ |  | BPDCN | 1 (0) | No |
| Biomarin | Talazoparib | Talzenna® | BRCA 1/2, HER2- | BC | 1 (1) | Yes |
| Epizyme | Tazemetostat | Taverik® | EZH2 | FL | 1 (1) | Yes |
| Wyeth | Temsirolimus | Torisel® |  | RCC and MCL | 2 (0) | No |
| GSK | Trametinib | Mekinist® | BRAF V600 | Melanoma and NSCLC | 2 (2) | Yes |
| Genentech | Trastuzumab | Herceptin® | HER2+ | BC and GC | 2 (2) | Yes |
| Seattle Genetics | Tucatinib | Tukysa® | HER2+ | BC | 1 (1) | Yes |
| AstraZeneca | Vandetanib | Caprelsa® | RET | MTC and NSCLC | 1 (1) | Yes |
| Roche | Vemurafenib | Zelboraf® | BRAF V600 | MM | 1 (1) | Yes |
| Abbvie | Venetoclax | Venclexta™ | TP53 | AML and CLL | 2 (1) | Yes |
| Roche/Genentech | Vismodegib | Erivedge® |  | BCC | 1 (0) | No |
| Merck | Vorinostat | Zolinza® |  | CTCL | 1 (0) | No |
| BeiGene | Zanubrutinib | Brukinsa™ |  | WM | 1 (0) | No |
| Regeneron | Ziv-Aflibercept | Zaltrap® |  | CRC | 1 (0) | No |

Legend: *Number in brackets is for number of indications where drug is used as precision medicine

ABL1 – breakpoint cluster region and Abelson murine leukemia; AdvSM - Advanced Systemic Mastocytosis; ALK -– Anaplastic lymphoma kinase; ALL - Acute Lymphocytic Leukemia; AML – Acute Myeloid Leukemia; BC - Breast Cancer; BCC - Basal Cell Carcinoma; BM - Bone Metastases; BPDCN - Blastic Plasmacytoid Dendritic Cell Neoplasm; BRAF- proto-oncogene B-Raf; BRCA -– breast cancer; CC – cervical cancer; CDx– companion diagnostic; CEL - Chronic Eosinophilic Leukemia; cHL – Classical Hodgkin's Lymphoma; CLL - Chronic Lymphocytic Leukemia; CML – Chronic Myeloid Leukemia; CRC – Colorectal Cancer; CSCC - Cutaneous Squamous Cell Carcinoma; CTCL - Cutaneous T-cell Lymphoma; DFSP - Dermatofibrosarcoma Protuberans; DLBCL - Diffuse Large B-cell Lymphoma; DTC - Differentiated Thyroid Cancer; EC - Endometrial Carcinoma; EGFR- epidermal growth factor receptor; EZH2 -enhancer of zeste homolog 2; FGFR -– fibroblast growth factor receptor; FL - Follicular Lymphoma; FLT3 -– fms like tyrosine kinase 3; GEJC - Gastroesophageal Junction Cancer; GIST - Gastrointestinal Stromal Tumor; HCC - Hepatocellular Carcinoma; HCL - Hairy Cell Leukemia; HER2 -– human epidermal growth factor receptor 2; HL - Hodgkin's Lymphoma; HNC - Head and Neck Cancer; HNSCC - Head and Neck Squamous Cell Carcinoma; IDH -– Isocitrate dehydrogenase; KIT – KIT proto-oncogene; MCC - Merkel Cell Carcinoma; MCD -multicentric Castleman’s disease; MCL - Mantle Cell Lymphoma; MDS/MPD - Myelodysplastic/Myeloproliferative Diseases; MET - MET gene; MF – Myelofibrosis; MM – Multiple Myeloma; MPM - Malignant Pleural Mesothelioma; MSI-H – Microsatellite Instability-High; MTC - Medullary Thyroid Cancer; NED – not enough data; NF1 - Neurofibromatosis Type 1; NR – not reported; NSCLC – Non-Small-Cell Lung Cancer; NT (GI or L) - Neuroendocrine Tumors of Gastrointestinal or Lung; NT (Pa) - Neuroendocrine Tumors of Pancreas; NTRK -– neurotrophin receptor tyrosine kinase; OC – Ovarian Cancer; OeC - Oesophageal Carcinoma; OSCC - Oral Squamous Cell Carcinoma; PaC – Pancreatic Cancer; PCTD – predates clinical trial data; Ph – Philadelphia chromosome; PDGFRA -– platelet-derived growth factor receptor A; PD-L1 – programmed death ligand 1; PIK3CA -– phosphatidylinositol-4,5-bisphosphate 3-kinase, catalytic subunit alpha; PrC – Prostrate Cancer; PV - Polycythaemia Vera; RAS -– rat sarcoma virus oncogene; RCC – Renal Cell Carcinoma; RET – rearranged during transfection; ROS -– c-ros oncogene; SCCHN - Squamous Cell Carcinoma Of Head and Neck; SCLC – Small-Cell Lung Cancer; SLL - Small Lymphocytic Lymphoma; SS - Sézary Syndrome; STS - Soft-Tissue Sarcoma; TNBC – Triple Negative Breast Cancer; TP53 -– tumor protein 53; UC - Urothelial Cancer; WM - Waldenström’s Macroglobulinemia.

**Table S3: Oncology medicines, their clinical trials, cost, and revenue**

| Launch year | Generic name | Biomarker? | | | Trial  duration (years) | Enrolees | Data quality grade | US$, millions | | | | | | |
| --- | --- | --- | --- | --- | --- | --- | --- | --- | --- | --- | --- | --- | --- | --- |
|  |  | Phase I | Phase II | Phase III |  |  |  | Revenue | R&D | POS | Capitalized* | Final | Tax  Rebate | Inflated  To 2020 |
| 2017 | **Abemaciclib** | Yes | Yes | Yes | 8 | 5,225 | C | 1,769 | 959 | 1,825 | 1,854 | 4,637 | 4,637 | 4,927 |
| 2017 | **Acalabrutinib** | No | No | No | 4 | 2,001 | C | 757 | 469 | 1,090 | 336 | 1,894 | 1,833 | 1,948 |
| 2013 | **Ado-trastuzumab** | Yes | Yes | Yes | 7 | 2,965 | C | 6,188 | 453 | 873 | 790 | 2,116 | 2,116 | 2,385 |
| 2013 | **Afatinib** | Yes | Yes | Yes | 10 | 6,429 | F | NR | NR | NR | NR | NR | NR | - |
| 2015 | **Alectinib** | Yes | Yes | Yes | 6 | 625 | D | 2,700 | 409 | 747 | 33 | 1,189 | 1,087 | 1,190 |
| 2019 | **Alpelisib** | Yes | Yes | Yes | 9 | 2,920 | C | 438 | 722 | 1,480 | 1,742 | 3,945 | 3,793 | 3,872 |
| 2016 | **Atezolizumab** | Yes | Yes | Yes | 8 | 7,741 | C | 5,188 | 559 | 1,304 | 1,195 | 3,058 | 2,940 | 3,183 |
| 2020 | **Avapritinib** | Yes | Yes | Yes | 5 | 903 | A | 21 | 677 | 782 | 153 | 1,613 | 1,362 | 1,362 |
| 2017 | **Avelumab** | Yes | Yes | Yes | 4 | 3,724 | C | 457 | 3,748 | 6,006 | 2,532 | 12,286 | 10,960 | 11,645 |
| 2012 | **Axitinib** | No | No | No | 10 | 4,138 | F | NR | NR | NR | NR | NR | NR | - |
| 2020 | **Belantamab** | No | No | No | 5 | 980 | C | 32 | 974 | 3,684 | 1,345 | 6,002 | 5,875 | 5,875 |
| 2004 | **Bevacizumab** | No | No | No | 8 | 1,064 | C | 68,399 | 489 | 897 | 146 | 1,532 | 1,532 | 2,073 |
| 1999 | **Bexarotene** | NR | NR | No | 5 | PCTD | D | NED | 224 | 441 | 508 | 1,173 | 1,173 | 1,762 |
| 2018 | **Binimetinib** | Yes | Yes | Yes | 9 | 3,719 | C | NED | 144 | 261 | 362 | 768 | 768 | 798 |
| 2014 | **Blinatumomab** | Yes | Yes | Yes | 11 | 809 | C | 1,335 | 917 | 2,286 | 2,710 | 5,913 | 5,578 | 6,170 |
| 2003 | **Bortezomib** | NR | NR | No | 8 | PCTD | C | 14,461 | 372 | 515 | 787 | 1,674 | 1,674 | 2,326 |
| 2012 | **Bosutinib** | Yes | Yes | Yes | 8 | 1,398 | E | 1,646 | NED | NED | NED | NED | NED | - |
| 2001 | **Brentuximab** | No | No | No | 5 | 550 | A | 4,207 | 279 | 864 | 915 | 2,058 | 1,904 | 2,737 |
| 2017 | **Brigatinib** | Yes | Yes | Yes | 6 | 420 | B | 251 | 491 | 1,107 | 573 | 2,172 | 1,986 | 2,110 |
| 2010 | **Cabazitaxel** | No | No | No | 11 | 838 | E | 4,652 | NR | NR | NR | NR | NR | - |
| 2012 | **Cabozantinib** | No | Yes | No | 7 | 1,968 | C | 3,713 | 461 | 965 | 1,381 | 2,807 | 2,807 | 3,219 |
| 2020 | **Capmatinib** | Yes | Yes | NR | 10 | 1,683 | C | 35 | 561 | 1,561 | 1,803 | 3,925 | 3,392 | 3,392 |
| 2012 | **Carfilzomib** | No | No | No | 7 | 2,375 | A | 6,033 | 502 | 1,156 | 648 | 2,307 | 2,307 | 2,646 |
| 2018 | **Cemiplimab** | Yes | Yes | Yes | 4 | 1,360 | A | 561 | 1,043 | 1,943 | 608 | 3,594 | 3,215 | 3,341 |
| 2014 | **Ceritinib** | Yes | Yes | Yes | 3 | 527 | C | 1,029 | 1,095 | 2,032 | 765 | 3,893 | 3,663 | 4,052 |
| 2004 | **Cetuximab** | Yes | Yes | Yes | 9 | 1,887 | A | 29,218 | 852 | 1,373 | 1,425 | 3,651 | 3,651 | 4,939 |
| 2015 | **Cobimetinib** | Yes | Yes | Yes | 9 | 2,856 | D | NED | 799 | 1,381 | 1,547 | 3,727 | 3,433 | 4,081 |
| 2017 | **Copanlisib** | Yes | No | No | 8 | 472 | F | NR | NR | NR | NR | NR | NR | - |
| 2011 | **Crizotinib** | Yes | Yes | Yes | 6 | 1,154 | E | 4,343 | NR | NR | NR | NR | NR | - |
| 2013 | **Dabrafenib** | Yes | Yes | Yes | 4 | 597 | E | 1,591 | NR | NR | NR | NR | NR | - |
| 2018 | **Dacomitinib** | Yes | Yes | Yes | 13 | 3,710 | F | NR | NR | NR | NR | NR | NR | - |
| 2016 | **Daratumumab** | No | No | No | 9 | 1,463 | E | 11,304 | NR | NR | NR | NR | NR | - |
| 2006 | **Dasatinib** | Yes | Yes | Yes | 2 | 2,209 | E | 19,252 | NR | NR | NR | NR | NR | - |
| 2015 | **Dinutuximab** | No | No | No | 5 | 56 | C | 498 | 54 | 161 | 37 | 252 | 252 | 276 |
| 2017 | **Durvalumab** | Yes | Yes | Yes | 4 | 8,256 | C | 4,220 | 4,273 | 6,506 | 2,399 | 13,178 | 12,622 | 13,410 |
| 2018 | **Duvelisib** | No | No | No | 7 | 1,612 | A | 30 | 868 | 2,152 | 672 | 3,692 | 3,374 | 3,506 |
| 2015 | **Elotuzumab** | No | No | No | 9 | 1,773 | B | 1,413 | 1,360 | 4,362 | 4,588 | 10,311 | 9,821 | 10,749 |
| 2017 | **Enasidenib** | Yes | Yes | Yes | 4 | 546 | A | 319 | 180 | 329 | 102 | 610 | 533 | 566 |
| 2018 | **Encorafenib** | Yes | Yes | Yes | 7 | 1,170 | C | 209 | 194 | 282 | 194 | 684 | 602 | 626 |
| 2019 | **Enfortumab** | No | No | No | 6 | 1,065 | C | 223 | 410 | 1,114 | 558 | 2,083 | 1,980 | 2,021 |
| 2019 | **Entrectinib** | Yes | Yes | NR | 5 | 773 | D | 22 | 451 | 1,107 | 524 | 2,082 | 1,910 | 1,950 |
| 2019 | **Erdafitinib** | Yes | Yes | Yes | 4 | 895 | F | NR | NR | NR | NR | NR | NR | - |
| 2004 | **Erlotinib** | Yes | Yes | Yes | 6 | 1,779 | C | 13,740 | 123 | 184 | 101 | 408 | 351 | 475 |
| 2009 | **Everolimus** | Yes | Yes | No | 9 | 5,470 | D | 14,315 | 1,853 | 3,663 | 6,660 | 12,175 | 12,175 | 14,698 |
| 2019 | **Fam-trastuzumab** | NR | NR | NR | 5 | NR | C | 48 | 2,341 | 5,339 | 2,330 | 10,009 | 9,424 | 9,621 |
| 2019 | **Fedratinib** | No | No | No | 12 | 191 | E | 60 | NR | NR | NR | NR | NR | - |
| 2003 | **Gefitinib** | NR | NR | NR | 6 | NR | E | 8,758 | NR | NR | NR | NR | NR | - |
| 2018 | **Gilteritinib** | Yes | Yes | Yes | 5 | 1,018 | C | 424 | 293 | 477 | 280 | 1,049 | 976 | 1,014 |
| 2018 | **Glasdegib** | No | No | No | 6 | 530 | F | NR | NR | NR | NR | NR | NR | - |
| 2013 | **Ibrutinib** | Yes | Yes | No | 5 | 1,620 | A | 32,610 | 784 | 1,694 | 389 | 2,867 | 2,578 | 2,906 |
| 2014 | **Idelalisib** | No | No | Yes | 6 | 1,756 | C | 825 | 851 | 2,200 | 1,166 | 4,218 | 3,906 | 4,321 |
| 2001 | **Imatinib** | NR | NR | No | 5 | NR | D | 65,324 | 292 | 846 | 506 | 1,644 | 1,583 | 2,275 |
| 2017 | **Inotuzumab** | No | No | No | 14 | 1,327 | E | 751 | NR | NR | NR | NR | NR | - |
| 2011 | **Ipilimumab** | Yes | Yes | Yes | 8 | 5,623 | E | 12,578 | NR | NR | NR | NR | NR | - |
| 2020 | **Isatuximab** | No | No | No | 10 | 1,759 | C | 55 | 3,338 | 6,664 | 5,986 | 15,987 | 15,821 | 15,821 |
| 2018 | **Ivosidenib** | Yes | NR | Yes | 4 | 789 | A | 196 | 444 | 775 | 311 | 1,529 | 1,360 | 1,413 |
| 2015 | **Ixazomib** | No | No | No | 8 | 2,471 | C | 2,305 | 1,604 | 3,463 | 2,848 | 7,916 | 7,515 | 8,225 |
| 2007 | **Lapatinib** | Yes | Yes | Yes | 5 | 4,011 | C | 3,111 | 478 | 792 | 420 | 1,690 | 1,628 | 2,019 |
| 2017 | **Larotrectinib** | Yes | Yes | NR | 5 | 204 | A | NR | 171 | 483 | 181 | 835 | 761 | 809 |
| 2015 | **Lenvatinib** | No | Yes | No | 10 | 2,816 | C | 3,925 | 1,754 | 3,763 | 4,351 | 9,868 | 9,429 | 10,320 |
| 2018 | **Lorlatinib** | Yes | Yes | Yes | 4 | 668 | E | 333 | NR | NR | NR | NR | NR | - |
| 2020 | **Margetuximab** | Yes | Yes | Yes | 10 | 1,215 | A | 5 | 543 | 926 | 1,173 | 2,641 | 2,641 | 2,641 |
| 2017 | **Midostaurin** | Yes | Yes | Yes | 15 | 1,517 | C | 852 | 1,369 | 2,319 | 4,862 | 8,550 | 8,262 | 8,778 |
| 2018 | **Mogamulizumab** | No | No | No | 12 | 1,065 | C | 273 | 835 | 2,525 | 3,735 | 7,095 | 6,886 | 7,155 |
| 2018 | **Moxetumomab** | No | No | No | 12 | 274 | C | 1 | 908 | 2,205 | 2,885 | 5,998 | 5,880 | 6,110 |
| 2020 | **Naxitamab** | NR | No | NR | 3 | 38 | C | 0 | 85 | 438 | 82 | 604 | 561 | 561 |
| 2017 | **Neratinib** | Yes | Yes | Yes | 14 | 6,180 | A | 649 | 1,089 | 1,580 | 2,565 | 5,233 | 5,233 | 5,560 |
| 2007 | **Nilotinib** | Yes | Yes | Yes | 3 | 548 | C | 17,395 | 534 | 1,257 | 267 | 2,058 | 1,946 | 2,413 |
| 2017 | **Niraparib** | No | No | Yes | 10 | 1,262 | A | 1,167 | 476 | 691 | 343 | 1,510 | 1,329 | 1,412 |
| 2015 | **Nivolumab** | Yes | Yes | Yes | 9 | 8,592 | E | 31,725 | NR | NR | NR | NR | NR | - |
| 2013 | **Obinutuzumab** | No | No | No | 6 | 5,033 | C | 1,833 | 1,255 | 2,991 | 1,864 | 6,111 | 5,658 | 6,377 |
| 2014 | **Olaparib** | Yes | Yes | Yes | 9 | 3,564 | C | 4,326 | 1,457 | 3,198 | 3,536 | 8,190 | 8,001 | 8,851 |
| 2015 | **Osimertinib** | Yes | Yes | Yes | 3 | 2,250 | C | 11,010 | 594 | 1,360 | 305 | 2,259 | 2,182 | 2,388 |
| 2015 | **Palbociclib** | Yes | Yes | Yes | 10 | 2,030 | E | 21,147 | NR | NR | NR | NR | NR | - |
| 2006 | **Panitumumab** | Yes | Yes | Yes | 5 | 1,796 | E | 7,090 | NR | NR | NR | NR | NR | - |
| 2009 | **Pazopanib** | No | No | No | 5 | 2,507 | C | 5,672 | 1,203 | 2,583 | 1,243 | 5,028 | 4,872 | 5,882 |
| 2014 | **Pembrolizumab** | Yes | Yes | Yes | 5 | 3,331 | E | 52,668 | NR | NR | NR | NR | NR | - |
| 2020 | **Pemigatinib** | Yes | Yes | Yes | 5 | 617 | C | 26 | 770 | 1,856 | 818 | 3,444 | 3,160 | 3,160 |
| 2012 | **Pertuzumab** | Yes | Yes | Yes | 15 | 10,808 | C | 14,345 | 924 | 2,315 | 7,682 | 10,922 | 10,922 | 12,527 |
| 2019 | **Polatuzumab** | No | No | No | 9 | 1,171 | C | 138 | 939 | 2,344 | 2,361 | 5,643 | 5,301 | 5,412 |
| 2013 | **Ponatinib** | Yes | Yes | Yes | 6 | 584 | A | 1,967 | 532 | 1,091 | 561 | 2,184 | 1,984 | 2,236 |
| 2020 | **Pralsetinib** | Yes | Yes | Yes | 3 | 309 | A | 1 | 260 | 506 | 168 | 934 | 829 | 829 |
| 2014 | **Ramucirumab** | No | No | No | 9 | 7,179 | D | 4,805 | 686 | 1,462 | 1,530 | 3,678 | 3,424 | 3,788 |
| 2012 | **Regorafenib** | No | No | No | 6 | 1,536 | D | 3,034 | 1,348 | 3,359 | 1,604 | 6,310 | 6,307 | 7,234 |
| 2017 | **Ribociclib** | Yes | Yes | Yes | 6 | 3,142 | C | 1,502 | 435 | 681 | 156 | 1,272 | 1,180 | 1,254 |
| 2020 | **Ripretinib** | Yes | NR | No | 5 | 583 | B | 39 | 349 | 553 | 178 | 1,080 | 943 | 943 |
| 1997 | **Rituximab** | No | No | No | 5 | NR | C | 90,201 | 312 | 1,447 | 595 | 2,355 | 2,232 | 3,439 |
| 2016 | **Rucaparib** | Yes | Yes | Yes | 7 | 1,400 | A | 469 | 525 | 938 | 653 | 2,115 | 1,917 | 2,075 |
| 2011 | **Ruxolitinib** | No | No | No | 4 | 1,038 | A | 15,534 | 415 | 1,546 | 650 | 2,611 | 2,452 | 2,866 |
| 2020 | **Sacituzumab** | Yes | Yes | Yes | 7 | 1,758 | C | 49 | 938 | 1,389 | 948 | 3,275 | 2,933 | 2,933 |
| 2019 | **Selinexor** | No | No | No | 7 | 3,553 | C | 8 | 624 | 1,817 | 1,336 | 3,777 | 3,621 | 3,697 |
| 2020 | **Selpercatinib** | Yes | Yes | Yes | 3 | 649 | C | 37 | 511 | 994 | 281 | 1,786 | 1,593 | 1,593 |
| 2020 | **Selumetinib** | No | Yes | Yes | 16 | 7,337 | C | 38 | 2,572 | 3,803 | 9,013 | 15,388 | 15,054 | 15,054 |
| 2014 | **Siltuximab** | No | No | No | 11 | 1,346 | F | NR | NR | NR | NR | NR | NR | - |
| 2015 | **Sonidegib** | No | No | NR | 6 | 1,031 | D | NR | 495 | 2,500 | 1,486 | 4,481 | 4,417 | 4,834 |
| 2005 | **Sorafenib** | No | No | No | 3 | 2,474 | E | 14,124 | NR | NR | NR | NR | NR | - |
| 2006 | **Sunitinib** | No | No | No | 3 | 1,104 | E | 16,353 | NR | NR | NR | NR | NR | - |
| 2020 | **Tafasitamab** | No | No | No | 7 | 777 | C | 22 | 356 | 836 | 624 | 1,816 | 1,813 | 1,813 |
| 2018 | **Tagraxofusp** | No | No | NR | 4 | 119 | A | 80 | 372 | 1,924 | 635 | 2,930 | 2,786 | 2,895 |
| 2018 | **Talazoparib** | No | Yes | Yes | 8 | 3,105 | C | NR | 740 | 1,116 | 1,079 | 2,935 | 2,662 | 2,766 |
| 2020 | **Tazemetostat** | Yes | Yes | Yes | 7 | 859 | A | 12 | 603 | 1,603 | 1,003 | 3,209 | 2,984 | 2,984 |
| 2007 | **Temsirolimus** | No | No | No | 11 | 4,093 | F | NR | NR | NR | NR | NR | NR | - |
| 2013 | **Trametinib** | Yes | Yes | Yes | 5 | 1,946 | C | 1,581 | 1,239 | 2,165 | 1,024 | 4,427 | 3,980 | 4,486 |
| 1998 | **Trastuzumab** | NR | Yes | NR | 5 | PCTD | D | 76,061 | 256 | 736 | 516 | 1,508 | 1,418 | 2,160 |
| 2020 | **Tucatinib** | Yes | Yes | Yes | 6 | 1,137 | A | 120 | 1,021 | 2,480 | 416 | 3,917 | 3,546 | 3,546 |
| 2011 | **Vandetanib** | No | Yes | NR | 6 | 667 | C | 652 | 596 | 1,714 | 954 | 3,264 | 3,186 | 5,985 |
| 2011 | **Vemurafenib** | Yes | Yes | Yes | 5 | 1,314 | C | 1,615 | 540 | 907 | 440 | 1,887 | 1,774 | 2,074 |
| 2016 | **Venetoclax** | No | Yes | No | 11 | 1,511 | C | 2,652 | 2,123 | 4,664 | 6,693 | 13,480 | 12,723 | 13,775 |
| 2012 | **Vismodegib** | No | No | No | 5 | 889 | C | 1,415 | 559 | 2,835 | 1,022 | 4,417 | 4,417 | 5,066 |
| 2006 | **Vorinostat** | No | No | No | 7 | 416 | F | NR | NR | NR | NR | NR | NR | - |
| 2019 | **Zanubrutinib** | No | No | No | 5 | 2,132 | A | 43 | 513 | 1,052 | 456 | 2,021 | 1,858 | 1,897 |
| 2012 | **Ziv-Aflibercept** | No | No | No | 9 | 4,808 | A | 1,761 | 1,439 | 4,357 | 4,667 | 10,463 | 10,463 | 12,000 |

Legend: *Costs were capitalized at a 10.5% real discount rate.

NED: not enough data; NR: not reported; PCTD: predates clinical trials; POS: probability of success; R&D: research and development

**Figure S1: Difference in research and development spend between precision and non-precision oncology medicines**


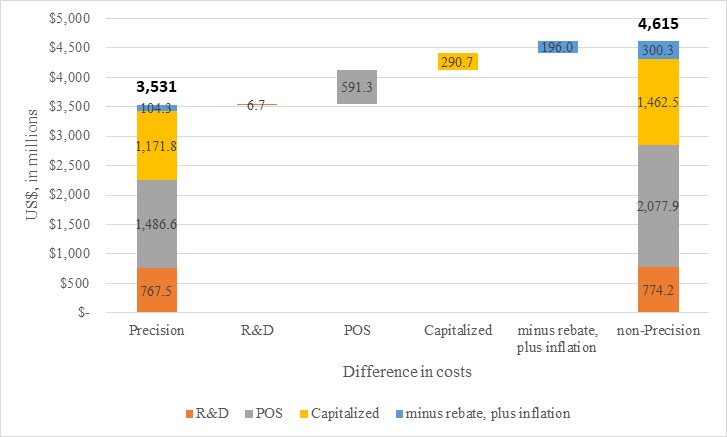


Legend: Precision versus non-precision oncology medicines’ R&D costs derived from SEC filings and annual reports, combined with the POS costs of completing clinical trials and the capitalized costs at an 10.5% real discount rate.

POS – probability of success; R&D – research and development

**Figure S2: Number of precision versus non-precision medicines launched from our sample**

**
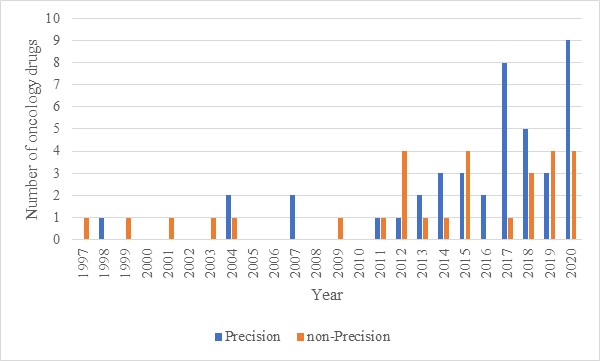
**

**References**

**1.** Wong CH, Siah KW, Lo AW. Estimation of clinical trial success rates and related parameters. *Biostatistics.* Apr 1 2019;20(2):273-286.
